# The JAK/STAT Pathway in Skeletal Muscle Pathophysiology

**DOI:** 10.3389/fphys.2019.00500

**Published:** 2019-04-30

**Authors:** Viviana Moresi, Sergio Adamo, Libera Berghella

**Affiliations:** ^1^ Unit of Histology and Medical Embryology, DAHFMO, University La Sapienza, Rome, Italy; ^2^ Interuniversity Institute of Myology, Rome, Italy; ^**3**^ Division of Biology and Biological Engineering, California Institute of Technology, Pasadena, CA, United States

**Keywords:** IL-6 cytokine, JAK/STAT pathway, skeletal muscle, organ cross talk, epigenetics

## Abstract

The Janus kinase (JAK)/signal transducer and activator of transcription (STAT) pathway is a key intracellular mediator of a variety of metabolically relevant hormones and cytokines, including the interleukin-6 (IL-6) family of cytokines. The JAK/STAT pathway transmits extracellular signals to the nucleus, leading to the transcription of genes involved in multiple biological activities. The JAK/STAT pathway has been reported to be required for the homeostasis of different tissues and organs. Indeed, when deregulated, it promotes the initiation and progression of pathological conditions, including cancer, obesity, diabetes, and other metabolic diseases. In skeletal muscle, activation of the JAK/STAT pathway by the IL-6 cytokines accounts for opposite effects: on the one hand, it promotes muscle hypertrophy, by increasing the proliferation of satellite cells; on the other hand, it contributes to muscle wasting. The expression of IL-6 and of key members of the JAK/STAT pathway is regulated at the epigenetic level through histone methylation and histone acetylation mechanisms. Thus, manipulation of the JAK/STAT signaling pathway by specific inhibitors and/or drugs that modulate epigenetics is a promising therapeutic intervention for the treatment of numerous diseases. We focus this review on the JAK/STAT pathway functions in striated muscle pathophysiology and the potential role of IL-6 as an effector of the cross talk between skeletal muscle and other organs.

## Introduction

The Janus kinase (JAK)/signal transducer and activator of transcription (STAT) pathway is a potent signaling cascade, evolutionarily conserved from flies to humans. It is upstream of multiple cellular activities such as proliferation, differentiation, migration, apoptosis, and cell communication or complex biological processes including inflammation, immune-system development, immune response, and cancer (Darnell et al., 1994; O’Shea et al., 2002, 2015; Bousoik and Montazeri Aliabadi, 2018). The JAK/STAT pathway was initially identified as responsive to interferon-gamma, although a variety of extracellular polypeptide signals and their transmembrane receptors were later found to activate it (Schindler et al., 1992; Heinrich et al., 1998; Aaronson and Horvath, 2002; O’Shea and Plenge, 2012).

In mammals, four members of the JAK proteins (JAK1, JAK2, JAK3, and TYK2) and seven members of the STAT family (STAT 1–4, STAT 5A/B, and STAT 6) were identified. They all share structurally and functionally conserved domains. JAK/STAT proteins are ubiquitously expressed, and different combinations of them respond to specific cytokines or growth factor signals, assuring a high degree of specificity with distinct *in vivo* roles (Aaronson and Horvath, 2002; Kisseleva et al., 2002; Rawlings et al., 2004). The mechanism of IL-6/JAK/STAT signaling cascade allows a direct communication between transmembrane receptors and the nucleus and can be summarized by the following steps: IL-6, the ligand, binds the IL-6r-Gp130 receptor complex and activates the JAK tyrosine kinases recruited to the intracellular domains of their receptors. Once activated, JAK proteins change their conformation, dimerize, phosphorylate, and activate their primary substrates, the STAT proteins. Tyrosine-phosphorylated STAT proteins homo- or hetero-dimerize and translocate to the nucleus, where they interact with coactivators and bind to specific regulatory elements in the promoter regions of thousands of different target protein-coding genes, along with microRNAs and long non-coding RNAs. STAT activity is regulated by phosphorylation, acetylation, and methylation, promoting STAT dimer stabilization, DNA binding, interaction with transcriptional coactivators, and target gene expression (Zuang, 2013; Yu et al., 2014; Zimmers et al., 2016). A further level of control is provided by negative regulators of JAK/STAT signaling that guarantee a cytokine-inducible feedback inhibition of signals from specific cytokine receptors (Greenhalgh and Hilton, 2001; Aaronson and Horvath, 2002; Linossi et al., 2013). JAK/STAT signaling operates also in response to IL-6 trans-signaling. Indeed, a soluble form of IL-6 receptor (sIL-6R), comprising the extracellular portion of the receptor, binds to IL-6, and the IL-6–sIL-6R complex is able to bind to and activate gp130 homodimers in cells which lack the membrane bound IL-6R (Kallen, 2002; Scheller et al., 2006). Thus, the JAK/STAT signaling cascade provides a remarkable direct and tuned translation of extracellular signals into a transcriptional response in a vast range of cells.

Primarily identified as functioning in hematopoietic cells, the JAK/STAT signaling cascade has been found to play a critical role in different cell types and tissues, including skeletal muscle. As skeletal muscle contracts, it secretes several cytokines into the circulation and the JAK/STAT pathway mediates the signaling of many of the myokines secreted by skeletal muscle (Pedersen and Febbraio, 2008; Hoffmann and Weigert, 2017).

Here, we will review the IL-6/JAK/STAT signaling cascade in myogenesis and skeletal muscle pathophysiology, focusing on its dichotomic role in myogenic cell proliferation and differentiation, as well as in muscle growth and muscle wasting. We will also discuss some examples of cross talk between muscle and other tissues. Finally, we will examine IL-6/JAK/STAT activity regulation, emphasizing the epigenetic mechanisms.

## IL-6/JAK/STAT Signaling Cascade in Skeletal Muscle

It is now widely accepted that through IL-6 family signals, the JAK/STAT pathway is required for efficient muscle fiber adaptation during development and regeneration. It was proposed that different combinations of the JAK/STAT pathway members have opposite effects on muscle differentiation and myogenesis. Indeed, the JAK1/STAT1/STAT3 axis promotes myoblast proliferation, preventing the premature differentiation into myotubes. Conversely, JAK2/STAT2/STAT3 induces myogenic differentiation, suggesting that other intracellular ligands act on JAK/STAT factors, to obtain distinct cellular responses at each step during development and myogenesis (Sun et al., 2007; Wang et al., 2008; Jang and Baik, 2013; Muñoz-Cánoves et al., 2013). Several studies demonstrated a role of the JAK/STAT pathway in regulating the myogenic progression of adult satellite cells (MuSCs), a population of cells that play a fundamental role in skeletal muscle postnatal growth and repair upon injury. MuSCs from IL-6 KO mice showed decreased proliferative capacity, both *in vivo* and *in vitro*. This impairment was caused by a lack of IL-6-mediated activation of STAT3 signaling. STAT3 induces the transcription of downstream genes involved in several biological functions, including myoblast proliferation, differentiation, and survival (Serrano et al., 2008; Toth et al., 2011). More recently, it has been shown that STAT3 knock-down (elicited by transient pharmacological or siRNA inhibition) in MuSCs, induced their expansion upon regeneration, but inhibited their differentiation, thus impairing muscle regeneration. Moreover, repeated intermittent administration of a STAT3 inhibitor in mdx mice, determined a sustained expansion of MuSC, contributing to an overall improvement in skeletal muscle repair (Tierney et al., 2014). Elsewhere, it was described that JAK2 or STAT3 KO in isolated MuSCs and pharmacological inhibition *in vivo* promoted symmetric satellite cell division and markedly improved their homing and repairing ability when transplanted into regenerating muscle (Price et al., 2014). However, different evidences were described when STAT3 depletion was investigated specifically by genetic deletion in MuSCs of mdx mice. By this approach, a progressive reduction of MuSC accompanied with aggravated fibrosis and muscle inflammation was observed. Then, a permanent knockout of STAT3 and a direct and long-term treatment with STAT3 inhibitors, which causes a gradual depletion of MuSCs, might have adverse effects on MuSCs and regeneration in DMD patients (Zhu et al., 2016), in contrast with other approaches such as transient inhibition by chemical inhibitors or siRNA, shown elsewhere (Price et al., 2014; Tierney et al., 2014). It may be speculated that transient and periodic reduction of STAT3 in cellular component of the MuSC niche, such as macrophages or fibro/adipogenic progenitors, known for playing an essential role in muscle regeneration (Bentzinger et al., 2013), is responsible for the beneficial effects observed in dystrophic muscle. Alternatively, IL-6 downstream effectors other than STAT3 could be active in MuSC in promoting muscle regeneration. Nevertheless, studies demonstrated that IL-6-mediated immunological responses may promote additional muscle fiber damage under conditions of dystrophin deficiency in mdx mice (Pelosi et al., 2015). Accordingly, IL-6 receptor blockade with the anti-IL-6 receptor antibody attenuated muscular dystrophy *via* promoting skeletal muscle regeneration in mdx and dystrophin-/utrophin-deficient mice (Pelosi et al., 2015; Wada et al., 2017).

IL-6/JAK/STAT pathway mediates increased proliferation of MuSC in other conditions such as acute exercise. Indeed, in a model of increasing mechanical loading, muscle hypertrophy resulted attenuated in IL-6 KO mice, due to an impaired MuSC proliferation and migration (Serrano et al., 2008). Moreover, mRNA expression for STAT3 target genes that regulate MuSC proliferation, migration, and differentiation was reduced (Serrano et al., 2008). Acute resistance exercise and resistance training activate the IL-6/STAT1/STAT3 signaling pathway in rat skeletal muscle (Begue et al., 2013) and in human (Trenerry et al., 2007, 2011), suggesting a potential role for STAT3 in the adaptive growth of skeletal muscle mediated by MuSCs. Nevertheless, more recent studies in human muscle biopsies and in STAT3 KO murine model concluded that STAT3 localized to the MuSCs is not required in load-induced skeletal muscle hypertrophy (Amorese and Spangenburg, 2017; Perez-Schindler et al., 2017). The cause of this contradictory evidence may be related to the methods for inducing hypertrophy and to the cell types where STAT3 activation occurs. Indeed, STAT3 activation in immune cells or other cells resident in skeletal muscle may also play significant roles in regulating muscle responses to exercise training (van de Vyver et al., 2016).

While sudden and acute induction of the IL-6 cascade promotes muscle growth, IL-6 sustained and elevated release and STAT3 activation have been associated with muscle atrophy occurrence in several catabolic conditions, such as obesity, diabetes, and age-induced sarcopenia or cancer (Zimmers et al., 2016). IL-6 overexpression in transgenic mice caused muscular atrophy, though entirely reversed by treatment with the membrane IL-6 receptor antibody (Tsujinaka et al., 1996). Interestingly, the negative role of IL-6 in the control of muscle mass was initially demonstrated using animal models of inflammation and cancer-associated cachexia. Cachexia is a muscle wasting syndrome accompanying many acute and chronic diseases, including cancer (Fearon et al., 2012; He et al., 2013; Argiles et al., 2014; Pigna et al., 2016). In cachexia experimental models, STAT3 expression is induced and correlates with increased expression of skeletal muscle ubiquitin E3 ligases. STAT3 dominant negative activity blocked the skeletal muscle loss downstream of IL-6, partly by inhibiting the activity of the ubiquitin proteasome system (UPS), *in vitro* and *in vivo* (Baltgalvis et al., 2009; Bonetto et al., 2011, 2012). Coherently, treatment with neutralizing antibodies prevented the increase of IL-6 concentration, exerting a protective effect on body weight loss in cachectic mice and blocking STAT3 activation reduced muscle wasting (Strassmann et al., 1992; Oldenburg et al., 1993; Haddad et al., 2005; Zimmers et al., 2016). Moreover, treatment of cachectic mice with pharmacological inhibitors of the JAK/STAT pathway components, partially prevented muscle mass loss (Gilabert et al., 2014; Pretto et al., 2015; Silva et al., 2015). JAK/STAT pathway activation is responsible for muscle atrophy by several potential mechanisms. In cachexia and chronic kidney disease models, both of which exhibit muscle mass loss, STAT3 initiated muscle wasting by stimulating CCAAT/enhancer binding protein (C/EBPδ) expression and activity, which in turn increased myostatin, MAFbx/Atrogin-1, and MuRF-1 expression in myofibers (Zhang et al., 2013). Direct UPS activation can be mediated by STAT3 or indirectly *via* caspase-3 activation (Silva et al., 2015) or dependent on FoxO transcription factors (Hutchins et al., 2013; Judge et al., 2014). Cachexia has been also associated with posttranslational modifications of JAK/STAT3 components, such as increased muscle phospho-Y705-STAT3 and increased STAT3 localization in myonuclei (Bonetto et al., 2011). Muscle catabolic profile may also be caused by the reduction in ribosomal protein kinase S6K1 phosphorylation and the increase of SOCS3 transcription, an inhibitor of the JAK/STAT pathway (Haddad et al., 2005). Nevertheless, others found that IL-6 does not stimulate muscle loss *per se* (Baltgalvis et al., 2008), thus supposing that other cytokines activate JAK/STAT, which triggers skeletal muscle proteolysis (Zhang et al., 2013). More recently, it was also shown that IL-6 trans-signaling works as a novel potent inducer of autophagy in myotubes inducing pathway that may be important in cancer cachexia development (Pettersen et al., 2017). Furthermore, IL-6 trans-signaling/STAT3 axis was identified as a therapeutic target in advanced cancer patients presenting cachexia (Miller et al., 2017).

In the muscle microenvironment, a JAK/STAT pathway contribution in catabolic conditions can be considered in relation to its role in promoting the expansion of the satellite cell pool *in vitro* and *in vivo*, impairing differentiation and muscle repair (He et al., 2013; Muñoz-Cánoves et al., 2013; Price et al., 2014; Tierney et al., 2014; Sala and Sacco, 2016; Zhu et al., 2016). Other than activation of STAT3 in MuSCs, secreted and elevated IL-6 levels and persistent STAT3 activation were observed in atrophic conditions in the fibro/adipogenic progenitors (FAPs), a population of cells resident in skeletal muscle, fundamental for muscular regeneration and inducible source of IL-6 (Joe et al., 2010; Madaro et al., 2018). IL-6/STAT3 signaling inactivation in FAPs counteracted muscle atrophy and fibrosis in mouse models of acute denervation and amyotrophic lateral sclerosis (ALS) (Gurney et al., 1994). This suggests an alternative IL-6/JAK/STAT-mediated mechanism, which induces muscle mass loss and represents a possible therapeutic target for neurogenic atrophy diseases (Madaro et al., 2018; Marazzi and Sassoon, 2018).

**Figure 1:**
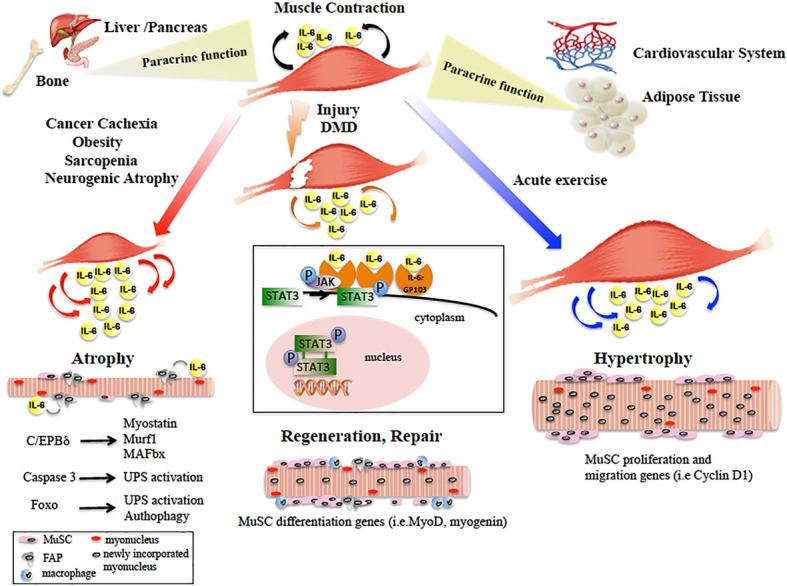
Diagram showing the main paracrine and dichotomic autocrine functions of the IL-6/JAK/STAT3 pathway in the pathophysiology of skeletal muscle. Skeletal muscle physiological contraction induces IL-6 release (black arrows), with paracrine effects on other organ metabolism. Upon injury or in DMD, IL-6 is released (orange arrows) following the inflammatory response and IL-6/JAK/STAT pathway promotes muscle repair by activating pro-myogenic genes (such as MyoD) that allow MuSC differentiation and fusion into new or existing myofibers. In catabolic conditions, IL-6 levels are elevated (red arrows) and induce muscle size loss, by activation of different pro-atrophic pathways in myofibers. In neurogenic atrophy, FAPs activate the IL-6/JAK/STAT pathway. In response to acute exercise, IL-6 is highly produced (blue arrows) and IL-6/JAK/STAT pathway is activated, inducing pro-proliferation and pro-fusion genes that control contribution of MuSC to myofiber growth. In the box, the IL-6/JAK/STAT3 signaling model is shown. IL-6 binds the IL-6r-Gp130 receptor complex and activates the JAK tyrosine kinases. Once activated, JAK proteins dimerize, phosphorylate, and activate their primary substrates, the STAT proteins. Phosphorylated STAT proteins dimerize and translocate to the nucleus, where they activate different target protein-coding genes.

Altogether, the autocrine and paracrine action of the IL-6/JAK/STAT pathway on skeletal muscle has opposite effects on satellite cells differentiation and proliferation, hence on muscle homeostasis. Moreover, it causes both deleterious (pro-atrophy) and beneficial (pro-repair and pro-growth) effects on muscle fiber size (Figure 1). The balance between these opposite outcomes may depend on the fine tuning of the JAK/STAT pathway. This effect can be mediated by the interaction of the JAK/STAT molecular effectors with the myofibers or with the multiple cell types of the muscle niche. Further studies will provide new insights to elucidate the molecular mechanism underlying this complex regulation.

## IL-6 As a Mediator of the Cross Talk Between Skeletal Muscle and Other Organs

Progressive discovery of new myokines by application of new technologies contributed to the definition of the muscle secretome and to provide new insights regarding their therapeutic potential in the treatment of obesity, metabolic disease, and cancer (Whitham and Febbraio, 2016).

IL-6 was the first cytokine to be proposed as a myokine (Pedersen et al., 2003), and the first myokine found to be secreted during exercise, playing important roles in regulating the metabolism of other organs (Goldstein, 1961). One of the main paracrine functions of IL-6 is to lead to an increase in hepatic glucose production, which works as an energy source for contracting muscles (Febbraio et al., 2004). Furthermore, during exercise, skeletal muscle performs also an “energy sensing” role, affecting some metabolic processes and, through IL-6, mediates the cross talk with insulin-sensitive tissues. By activation of AMP-activated protein kinase (AMPK) and/or PI3-kinase, IL-6 leads to enhanced glucose uptake, lipolysis and fatty acid oxidation, which provide energy from skeletal muscle (Keller et al., 2001; Al-Khalili et al., 2006). Moreover, IL-6/JAK/STAT plays also a major role in mediating communication between skeletal muscle and pancreas, enhancing glucose tolerance by activating glucagon-like peptide 1 (GLP1) in pancreatic islets. This allows adaption to changes in insulin demand, reduction of food intake and body weight, though having a role in improving metabolic homeostasis in obesity and type 2 diabetes (Bouzakri et al., 2011; Plomgaard et al., 2012; Ellingsgaard et al., 2015).

A direct cross talk between muscle tissue and adipose tissue (AT) occurs in obese mice. In this condition, subcutaneous adipose tissue does not contribute to IL-6 secretion during exercise, so the increased IL-6 produced following prolonged exercise probably derives from skeletal muscle (Eder et al., 2009). In fact, obese mice exposed to acute exercise showed an IL-6 induction, accompanied by increase in STAT3 phosphorylation, reduction in M1 macrophages, and inflammation in infiltrates in AT (Macpherson et al., 2015).

Skeletal muscle-derived growth factors and cytokines have a critical role in maintaining the cardiovascular system. The trophic cascade initiated by skeletal muscle JAK/STAT3 signaling increases growth factor levels in multiple tissues, leading to elevated circulating HGF and VEGF. Their synergistic actions further activate the myocardial repair mechanisms orchestrated by PI3K-AKT, ERK (Shabbir et al., 2010). The cardioprotective events of the IL-6/JAK/STAT3 apparently contradict its activity in promoting skeletal muscle wasting, underlying the multiple role of JAK-STAT3 signaling in different tissues.

It is known that the myokines mediate direct communication from skeletal muscle to bone. Elevated IL-6 induces bone loss in IL-6 KO mice (De Benedetti et al., 2006) and is a systemic mediator of the bone loss in dystrophy. In this context, elevated levels of IL-6 produced by inflamed skeletal muscle induce osteoclast increase, which can be reduced by treatment with an anti-IL-6 antibody (Rufo et al., 2011). Interestingly, IL-6, by affecting the functions of liver, fat, and intestine, induces secretion of hepatokines and adipokines to regulate bone formation and bone resorption (Rufo et al., 2011; Guo et al., 2017).

Collectively, these data show that IL-6 produced by contracting skeletal muscle plays important roles in regulating metabolism in other organs (Figure 1). Hence, lack of physical activity appears to affect a whole network of organs such as liver, pancreas, fat, and bone. From this perspective, the IL-6/JAK/STAT pathway is nodal in novel therapeutic approaches for the preventive treatment of diseases including cardiovascular diseases, type 2 diabetes, cancer, and osteoporosis.

## Epigenetic Control of IL-6/JAK/STAT Pathway

**Figure 2:**
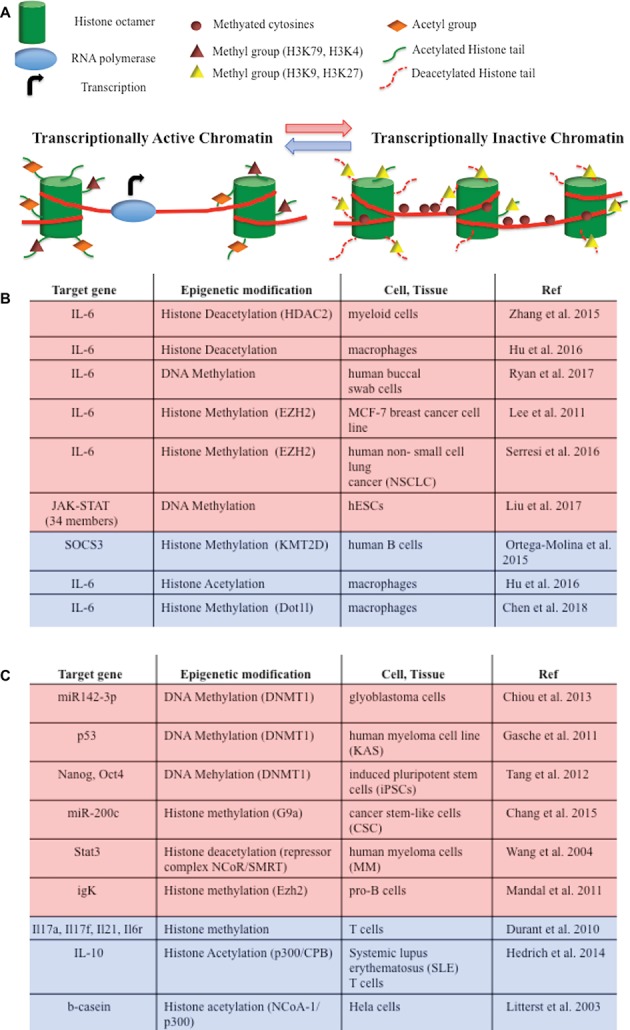
**(A)** DNA methylation and histone modifications are involved in epigenetic modulations of IL-6/JAK/STAT pathway members. They induce chromatin conformational transitions, altering accessibility of the transcriptional machinery (transcriptional active chromatin—blue arrow; transcriptional inactive chromatin – red arrow). DNA methylation is a process by which methyl groups are added to the cytosine of the DNA molecule and acts to repress gene transcription. Histone acetylation transfers acetyl groups to the histones and increases gene expression. Histone deacetylation removes acetyl groups from histones, allowing the histone to wrap more tightly the DNA and preventing transcription. Histone methylation adds methyl groups to the amino acids of the histones. Methylation of histones can either increase (i.e., H3K79, H3K4) or decrease (i.e. H3K9, H3K27) gene transcription. **(B)** Epigenetic modifications of IL-6/JAK/STAT pathway member genes that lead to gene repression (red) or gene activation (blue). **(C)** Epigenetic switches involving IL-6/JAK/STAT pathway members that lead to gene repression (red) or gene activation (blue) in tumorigenesis and development.

Considering that IL-6 mediates cellular response to stress or metabolic changes, it is not surprising that the IL-6 pathway is also modulated at the epigenetic level, at least by two main mechanisms, i.e., DNA methylation and histone modifications (Figure 2A). IL-6 gene transcription itself is directly modulated by histone acetylation and methylation in macrophages and in cancer cell lines (Lee et al., 2011; Zhang et al., 2015; Hu et al., 2016; Serresi et al., 2016; Chen et al., 2018). Moreover, an association between IL6/JAK/STAT DNA altered methylation and depression has been recently described (Ryan et al., 2017). JAK and STAT gene hypomethylation might also exert influences on erythroid lineage choice by specifically upregulating erythropoiesis transcription factors (Liu et al., 2017). In B cells activating pathway, the lysine-specific histone methyltransferase KMT2D affected H3K4 methylation and expression of a specific set of JAK-STAT genes (Figure 2B; Ortega-Molina et al., 2015).

Interestingly, IL-6 signaling modulates or cooperates with epigenetic mechanisms in regulating chromatin accessibility in tumorigenesis and development (Figure 2C). For instance, IL-6 promotes hypermethylation of the miR142-3p promoter in glioblastoma cells and of certain tumor suppressor genes in oral squamous cell carcinoma (Gasche et al., 2011; Chiou et al., 2013). IL-6-induced hypermethylation and gene silencing are mediated by DNA methyltransferases (DNMTs). IL-6 contributes to tumor growth by increasing DNMT expression and epigenetically repressing tumor suppressor genes or several microRNA in cancer cell lines (Hodge et al., 2005; Zhang et al., 2005, 2006; Braconi et al., 2010; Takeuchi et al., 2015). IL-6 also promotes DNA methylation of the promoter-bound STAT3, leading to a decrease in STAT3 DNA binding in human colon cancer cells (Yang et al., 2010), or of the *Foxp3* gene, thus influencing regulatory T cell development (Lal et al., 2009). T cells differentiation is also regulated by STAT3-dependent histone trimethylation at target gene loci (Durant et al., 2010).

STAT proteins have also been implicated in epigenetic switches involving somatic cell and metabolic reprogramming, inflammation, and transformation. JAK/STAT3 activity plays a fundamental role in facilitating DNA demethylation/*de novo* methylation to complete reprogramming of pre-iPSC (Tang et al., 2012). Both STAT3 and STAT5 mediate trans-activation and epigenetic remodeling of IL-10 through their interaction with the histone acetyltransferase p300 in lupus T cells (Hedrich et al., 2014). Furthermore, STAT proteins can recruit and form a repressor complex with either the histone methyltransferases, or with NCoR associated with histone deacetylases, repressing the transcription of genes or microRNA promoters (Nakajima et al., 2001; Litterst et al., 2003; Wang et al., 2004; Mandal et al., 2011; Chang et al., 2015).

To date, no evidence about the epigenetic control of IL-6 pathway has been reported in skeletal muscle. Identification of the epigenetic mechanisms regulating IL-6 gene expression, or the expression of the IL-6 pathway downstream effectors, as well as STAT protein interaction studies in specific muscle cell types or during muscle differentiation and their effects on muscle cell biology remain puzzling.

## Conclusion and Perspectives

The IL-6/JAK/STAT signaling cascade plays a dominant role in skeletal muscle pathophysiology. IL-6 autocrine, paracrine, and endocrine functions assign to its downstream effectors pivotal importance in skeletal muscle-wasting-associated diseases and other multiple system diseases where muscle acts in communication with other organs. Targeting the components of the JAK/STAT pathway recently emerged as a strategic approach for the treatment of inflammatory diseases and human cancer.

This review highlights the opposite outcomes on muscle biology caused by the amount of local and systemic release of IL-6. Transient release and short-term acute action have positive effects, by increasing the source of progenitors for regeneration and growth in skeletal muscle. This also affects metabolic processes in other organs, since it stimulates glucose production. In different circumstances, chronically elevated levels of IL-6 have negative consequences, promoting muscle atrophy through different mechanisms not completely yet elucidated. These antithetical effects can also be a key to the several discrepancies observed with different experimental approaches aimed to decipher the IL-6/JAK/STAT role in skeletal muscle functions. Moreover, the different cell and tissue compartments where IL-6 is produced and acts can account for the conflicting effects observed on muscle repair, growth, and wasting. Additionally, a role in these dichotomous outcomes can also be carried out by the combined action of the IL-6 trans-signaling, which is pro-inflammatory and the classic IL-6 signaling *via* the membrane bound IL-6R, which instead is needed for regenerative or anti-inflammatory activities of the cytokine (Scheller et al., 2011; Rose-John, 2012; Belizário et al., 2016).

Development of specific inhibitors or neutralizing antibodies against IL-6/JAK/STAT pathway factors may be proposed for diseases that cause muscle wasting, including DMD, cancer cachexia, and diabetes. Indeed, many studies demonstrated that they could ameliorate muscle wasting in mice (Zhang et al., 2013; Pretto et al., 2015; Silva et al., 2015). Nevertheless, they can act by nonspecific mechanisms and on cells and tissues other than myofibers.

In the light of this evidence, any therapeutic approach for skeletal muscle-wasting diseases targeting IL-6/JAK/STAT pathway should ideally consider the rate and the site of IL-6 production, in order to promote the benefits and avoid the detrimental effects.

Future studies on the mechanisms of action underlying the IL-6/JAK/STAT signaling cascade will provide new insights to tailor therapeutic strategies for each physiopathological condition. Further investigation of epigenetic mechanisms regulating and involving IL-6/JAK/STAT signaling cascade may identify epigenetics modification of IL-6 and its effectors as biomarkers of several diseases. Moreover, the IL-6/JAK/STAT molecular factors may represent new targets of the evolving epigenetics therapies directed to systemic pathologies and neuromuscular diseases, where combinations of epigenetic modulators may provide a tool to discriminate among alternative therapeutic effects.

## Author Contributions

All authors listed have made a substantial, direct and intellectual contribution to the work, and approved it for publication.

### Conflict of Interest Statement

The authors declare that the research was conducted in the absence of any commercial or financial relationships that could be construed as a potential conflict of interest.

## Abbreviations

AT: Adipose tissue
C/EBPδ: CCAAT/enhancer binding protein
DMD: Duchenne muscular dystrophy
DNMT: DNA methyltransferase
FAP: Fibro-adipogenic progenitors
FoxO: Forkhead box
IL-6: Interleukin 6
JAK: Janus kinase
MAFbx: Muscle atrophy F-box
MuRF: Muscle RING finger
MuSC: Muscle satellite cell
sIL-6R: Soluble interleukin 6 receptor
STAT: Signal transducer and activator of transcription
UPS: Ubiquitin proteasome system
